# Role of Cardiac Biomarkers in the Evaluation of Rheumatoid Arthritis: A Systematic Review

**DOI:** 10.7759/cureus.47416

**Published:** 2023-10-21

**Authors:** Saatvika R Borra, Binay K Panjiyar, Sourav S Panicker, Anirudh Danduboyina

**Affiliations:** 1 Internal Medicine, Jawaharlal Nehru Medical College, Belagavi, IND; 2 Global Clinical Scholars Research Training (GCSRT) Post Graduate Medical Education (PGMEE), Harvard Medical School, Boston, USA; 3 Internal Medicine, California Institute of Behavioral Neurosciences and Psychology, Fairfield, USA; 4 Internal Medicine, Dr. D. Y. Patil Medical College, Hospital and Research Centre, Pune, IND; 5 Internal Medicine, Gandhi Medical College, Hyderabad, Telangana, IND

**Keywords:** medical field, cardiovascular system, evaluation, rheumatoid arthritis, cardiac biomarkers

## Abstract

Rheumatoid arthritis (RA) is a chronic inflammatory disease that can cause permanent joint damage and premature death. Cardiovascular disease (CVD) has recently been known to have become a significant cause of death in rheumatoid arthritis patients, and cardiovascular (CV) deaths have risen by 20-50% in rheumatoid arthritis patients. Early detection methods are necessary to improve the outcome for such patients. Cardiac biomarkers have been proven to be an effective tool for evaluating the heart's activity. In this study, we have used a systematic literature review approach in order to establish an overview of the current literature, highlight the advantages of using cardiac biomarkers in early detection and diagnosis, and improve the prognosis of patients with rheumatoid arthritis. We reviewed 269 articles from January 1, 2012, to August 6, 2023, from reputed journals, out of which we focused on seven papers for in-depth analysis. This analysis considered certain factors, including the age factor, sex factor, clinical risk score, and comparison of the benefits of using this method amongst clinicians for diagnosis purposes. The systematic review has revealed that cardiac biomarkers have a good ability to act as predictors of subsequent cardiovascular events. Cardiac biomarkers include high-sensitivity troponin T (hsTropT) and B-type natriuretic peptide (BNP). We learned that the cardiac biomarkers indicate inflammation, extracellular matrix remodeling, congestion, and myocardial injury, which are linked with elementary changes in cardiac structure and function. Biomarkers could be used for the purpose of screening cardiac variations in patients with rheumatoid arthritis. However, this method tends to have its own challenges to implement, considering other factors such as age and NSAID use. Nonetheless, further research and intervention about the use of cardiac biomarkers are important in order to earn the potential to make this method available to be used worldwide to improve outcomes for patients with rheumatoid arthritis.

## Introduction and background

Rheumatoid arthritis (RA) is a chronic inflammatory disease known to cause joint deformity, disability, and premature death. Several studies from the past 10 years have shown reports of an increase in mortality risk in patients with rheumatoid arthritis. Recent studies have proven that this increased risk may be attributed mainly to cardiovascular (CV) events. The overall mortality rate has been increasing among RA patients. RA patients have a greater chance of developing silent ischaemic heart disease and experiencing sudden death. These patients have a higher chance of developing inflammation and unstable atherosclerotic plaques in their coronary arteries. Rheumatoid arthritis patients are not just at high risk of developing heart failure; they also have a greater tendency to die shortly after heart failure. Therefore, it is essential to pay more attention to the cardiovascular risks associated with rheumatoid arthritis patients in order to reduce cardiovascular morbidity and mortality among rheumatoid arthritis patients. The cardiovascular risk factors need to be assessed in order to control them and prevent the increasing mortality rates among rheumatoid arthritis patients [1]. Recent studies have shown that rheumatoid arthritis patients with heart failure cannot be explained by traditional cardiovascular risk factors alone [2].

Cardiovascular diseases (CVD) are estimated to be a major cause of death worldwide. Cardiovascular disease, according to the World Health Organization (WHO), causes nearly 17.9 million deaths a year, which accounts for 32% of all deaths. Rheumatoid arthritis patients are known to be more than twice at risk of developing a myocardial infarction when compared to the general population [3]. It is significant to research such chronic disorders in order to study the biomarkers that contribute to potential risks.

Rheumatoid arthritis is a chronic inflammatory disease. It is one of the most common types of chronic inflammatory disease known to affect the joints. This could lead to permanent joint damage and disability. Rheumatoid arthritis is an autoimmune systemic inflammatory disease that occurs due to the action of genetic and environmental factors that lead to a result of a cascade of immune reactions, leading to the development of synovitis, joint damage, and structural bone damage [4]. This further causes pain, disability, and emotional, social, and economic challenges [5]. There is an increased risk of the cardiovascular system in RA patients due to increased levels of inflammatory markers such as CRP, erythrocyte sedimentation rate, rheumatoid factor, and anticitrullinated protein antibodies [6]. Traditional risk factors like smoking, arterial hypertension, dyslipidemia, insulin resistance, and obesity are commonly seen in patients with rheumatoid arthritis. It has been noted that a range of disease-associated single nucleotide polymorphisms (SNPs) are seen in both RA and CVD. Human leukocyte antigens (HLAs) play a role in the pathogenesis of RA, and HLA-DRB1*04 is also an important risk factor for RA and CVD. Some independent genetic forms of inflammatory mediators are also frequent risk factors for RA and CVD [3].

Rheumatoid arthritis is known to be caused by genetic and environmental factors. Genetic variation among twins has shown a 50-60% risk for the development of rheumatoid arthritis. HLA-DRB1*01, *04, and *10 alleles have shown a very strong genetic risk factor for the development of rheumatoid arthritis. Smoking has been identified as the most substantial environmental risk factor for rheumatoid arthritis. Recent advances have shown that there is also a role for the oral and gut microbiomes in the development of rheumatoid arthritis. Among the RA patients, 50-80% are known to have autoantibodies. This has helped in the identification of the subgroups of RA: APCA positive and APCA negative [7].

Rheumatoid patients with atherosclerosis and increased intima-media thickness (IMT) were measured by ultrasonography, which was known to be an early indicator of generalized atherosclerosis. This ultrasound indicator was, however, not easily accessible and had disadvantages such as failure to detect the very early phases of the development of atherosclerosis. In order to enhance the benefits of health and cost-effectiveness, it became crucial to improve the accuracy of the identification of patients at risk for increased atherosclerosis and, subsequently, cardiovascular death. There has recently been a development in the identification of biomarkers that could enhance the prediction of cardiovascular events and death [8,9].

Cardiac biomarkers in the system can help in the evaluation of the cardiovascular system in patients with rheumatoid arthritis. Cardiac biomarkers include N-terminal pro-brain natriuretic peptide (NT-proBNP), troponin, creatinine, etc. Cardiac biomarkers help to provide important information regarding the underlying pathophysiology processes in patients with rheumatoid arthritis as well as cardiac alterations, even when they are asymptomatic. By determining the biomarkers that are circulating in the system, one can understand the structure and function of the heart in patients with RA, and this could help to recognize the patients who are at risk of developing symptomatic heart failure, and we can use these cardiac biomarkers to implement a better screening strategy [10].

It has been noted that there has been an increase in the trend of myocardial infarction, arrhythmia, pericardial disease, and left ventricular (LV) dysfunction in rheumatoid arthritis patients compared to the prevalent population. It has also been noted that patients with an active disease have worse left ventricular systolic and diastolic function. Cardiac biomarkers such as high-sensitivity troponin T (hsTropT) and B-type natriuretic peptide (BNP) have shown evidence to detect atypical heart structure and function. The cardiac biomarkers hsTropT and BNP are also found in patients with hypertrophic cardiomyopathy; this could be explained by persistent cardiac injury that elevates the cardiac biomarker levels in malignant LV hypertrophy and subclinical cardiac injury in the prevalent population. Reports have also shown that cardiac troponin and BNP levels rise in patients with myotonic dystrophy or chronic kidney disease, as well as in the subclinical general population with atypical LV systolic and diastolic functions. Cardiac troponin has been indicated to be associated with inert myocardial injury in rheumatoid arthritis patients [11]. Highly sensitive cardiac troponin (HS-cTn) has proven to hold a high prognostic value in several conditions in patients with acute myocardial infarction, chest pain, and heart failure in low-risk groups of people. The NT-proBNP has been known to indicate advanced cardiac mortality and morbidity in the common population and in patients with heart failure, as well as stable coronary heart disease [2]. It was noted in a study that NT-proBNP, ox-LDL, and anti-Apo A-I are crucial indicators of cardiovascular events. Anti-apolipoprotein A-I was known to significantly play an important role in improving the prognostic ability of the Framingham 10-year cardiovascular risk score (FRS) [12]. It was noted that in patients with RA who had an increase in inflammation, they presented with a decrease in routinely measured lipids, including LDL cholesterol, and an increase in markers of subclinical myocardial injury was observed. This further shows the divergence in biomarkers of CVD risk and enhances the benefit of including hs-cTnT for CVD risk stratification in patients with rheumatoid arthritis [13].

This systemic review evaluated the availability of evidence for the effectiveness of using cardiac biomarkers in patients with rheumatoid arthritis by answering the question: Can the evaluation of rheumatoid arthritis based on the role of cardiac biomarkers be done?

## Review

Methods

**Figure 1:**
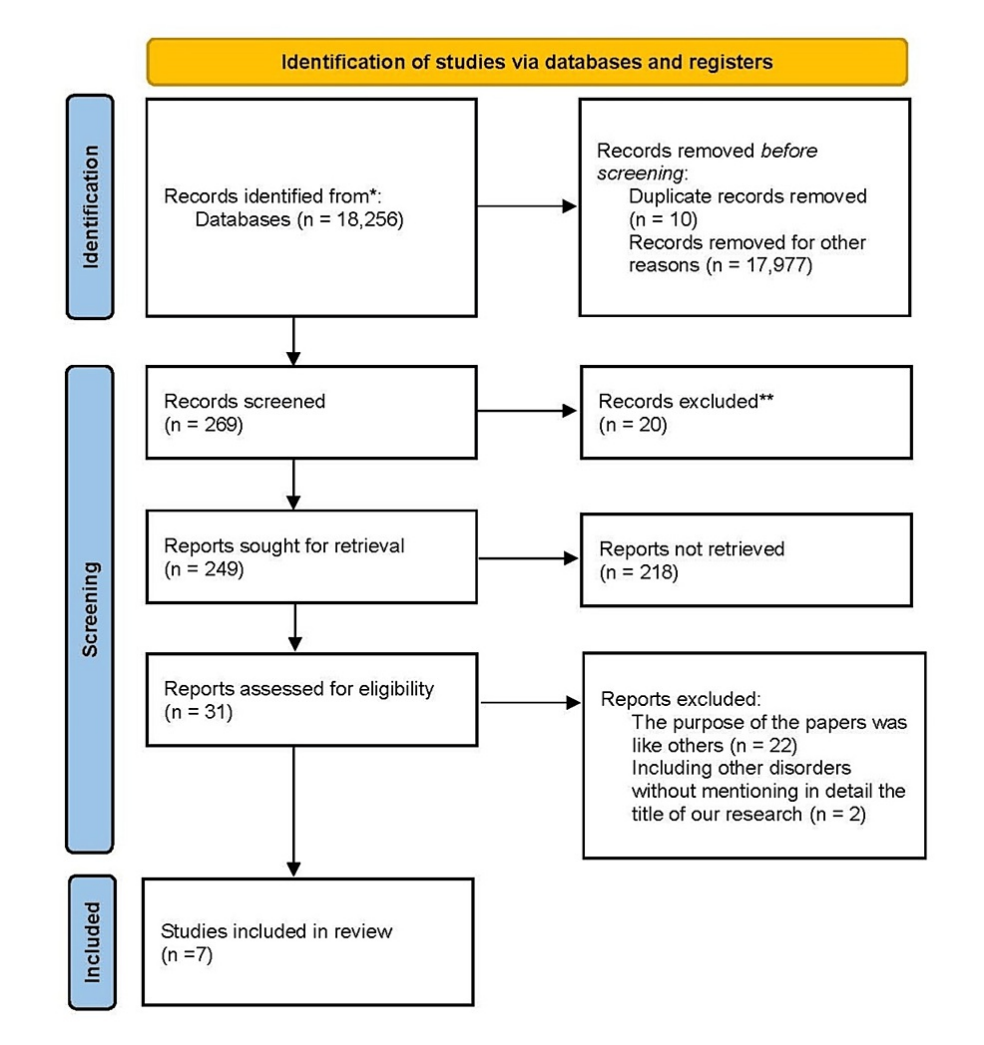
PRISMA flow diagram demonstrating the search strategy and study selection procedure for the systematic review. PRISMA: Preferred Reporting Items for Systematic Reviews and Meta-Analyses.

The review targets clinical studies regarding the use of cardiac biomarkers in the evaluation of rheumatoid arthritis. We excluded animal studies. The review is in accordance with the guidelines for Preferred Reporting Items for Systematic Reviews and Meta-Analyses (PRISMA) for 2020 in Figure 1 and only utilizes data collected from published papers, disposing of the requirement for ethical approval.

Systemic Literature Search and Study Selection

We conducted an in-depth search for appropriate publications using PubMed, including Medline and Google Scholar. We looked for studies done in review papers, editorials, and commentaries on PubMed. However, we kept searching for additional studies that fulfilled our inclusion criteria.

We had a record of abstracts that we accurately reviewed for inclusion using particular criteria. The criteria involved using methods targeting cardiovascular applications and distinctly described the clinical cohort in the study. We excluded review papers and animal studies.

Inclusion and Exclusion Criteria

We established particular criteria for including and excluding participants to attain our study goals. Our criteria can be referred to in Table 1.

**Table 1:** Showing the criteria adopted through the literature search procedure

	Inclusion criteria	Exclusion criteria
(a)	Human studies	Animal studies
(b)	From 2012 to 2023	Only methodological studies explaining programming details
(c)	English text	Non-English text
(d)	Gender: All	
(e)	Age: +19 years	Age: <19 years
(f)	Free papers	Papers requiring purchasing
		Studies including clinical data more than cardiovascular diseases

Search Strategy

The population, intervention/condition, control/comparison, and outcome (PICO) criteria were employed to carry out a detailed literature review. The search was done on databases such as PubMed (including Medline) and Google Scholar Libraries, utilizing applicable keywords such as rheumatoid arthritis, cardiac biomarkers, evaluation, and cardiovascular system. The medical subjects heading (MeSH) approach for PubMed (including Medline) and Google Scholar, as mentioned in Table 2, was undertaken to develop a comprehensive search strategy.

**Table 2:** Exhibiting search strategy, search engines utilized, and the number of outcomes presented.

	Database	Search strategy	Search results
(a)	PubMed	Rheumatoid arthritis, cardiac biomarkers, or cardiovascular systems and evaluation (Date: January 01, 2012 to June 08, 2023)	256
(b)	Google Scholar	Rheumatoid arthritis and cardiac biomarkers or cardiovascular system and evaluation	18,000

Quality Appraisal

To verify the credibility of our chosen papers, we have used several quality evaluation tools. We utilized the PRISMA checklist and Cochrane bias tool assessment for randomized control trials, systemic reviews, and meta-analyses. Non-randomized clinical trials were assessed using the Newcastle-Ottawa tool scale. We evaluated the quality of the qualitative studies, as displayed in Table 3, utilizing the critical appraisal skills program (CASP) checklist. To avert any confusion in the classification, we used the scale for the assessment of narrative review articles (SANDRA) to evaluate the article’s quality.

**Table 3:** Displaying quality appraisal tools used. PRISMA: Preferred reporting items for systemic reviews and meta-analyses; SANDRA: Scale for the assessment of non-systematic review articles.

Quality appraisal tools used	Types of studies
Cochrane bias tool assessment	Randomized control trials
Newcastle-Ottawa tool scale	Non-RCT and observational studies
PRISMA checklist	Systemic reviews
SANDRA checklist	Any other without clear method section

Results

After searching through three selected databases, PubMed, Medline, and Google Scholar, we then accurately reviewed each paper and claimed specific criteria, which led to obtaining 18,256 articles. From these articles, we decided not to use 17,987 of them due to undesirable titles and abstracts. We precisely evaluated the remaining 269 articles and excluded 262 more as their content did not match our inclusion criteria. Finally, we carried out an elaborate quality check on the remaining seven papers, which had met all our criteria. These seven articles are included in our final systemic review. Table 4 provides a detailed description of each.

**Table 4:** Summary of results of selected papers. RA: rheumatoid arthritis; FRS: Framingham 10-year cardiovascular risk Score; CV: cardiovascular; HS-cTnT: high-sensitive cardiac troponin T; NT-proBNP: N-terminal pro-brain natriuretic peptide; BNP: brain natriuretic peptide.

Author/year	Country	Study design	Database used	Objective
Avouac et al. [2]	Paris, France	Cross-sectional study	PubMed and Medline	To measure the levels of HS-cTnT and NT-proBNP and examine if it correlates
Popescu et al. [3]	Romania	Systemic review with meta-analysis	Google scholar	Studies evaluating early detection of atherosclerosis to reduce CV risk in RA patients
Ambrosino et al. [9]	Napoli, Italy	A systemic review with meta-analysis	PubMed and Medline	Studies to evaluate the relationship between RA and markers of cardiovascular risk
Kobayashi et al. [10]	Portugal	Cohort study	PubMed and Medline	To study the correlation amongst circulating biomarkers and echocardiographic parameters in RA patients
Aiewruengsurat et al. [11]	Thailand	Cross-sectional study	PubMed and Medline	To assess the correlation between cardiac and rheumatoid biomarkers levels with cardiac structure and function
Finckh et al. [12]	Geneva, Swizerland	Cohort study	PubMed and Medline	To determine whether adding FRS would enhance the CV prognostic validity in RA
Weber et al. [13]	Atlanta, USA	Cohort study	Google Scholar	To determine the association of RA patients with concominant temporal patterns in markers of myocardial injury

Discussions

There has been an increase in the risk of mortality in a large part of the population with RA, associated with an increased risk of CV death. Early detection and treatment are crucial for survival [1]. In RA patients, risk stratification is a challenging problem as the traditional risk factors tend to overestimate or underestimate the CV risk [14]. The Framingham 10-year risk score for CHD risk assessment for diagnosis, evaluation, and treatment of high blood cholesterol in adults has been practiced in the United States [15]. Several other algorithms, such as the sex-specific and race-specific pooled cohorts (SCORE) and the QRESEARCH cardiovascular risk algorithm (QRISK), can be used as well to identify the predictive factors. These models tend to have their own disadvantages, like differences among validation cohorts and exaggerating the risk of CVD. Such models also must pay more attention to the prime variables, resulting in less valid predictions. For patients with rheumatoid arthritis, better advanced predictive tools would be necessary [16].

Cardiac biomarkers could be used to sort out cardiac abnormalities in RA patients. The RA cohort provides us with a great opportunity to investigate the cardiac biomarkers linked to echocardiographic parameters in RA and the prognostic value of echocardiographic parameters. Moreover, the link between biomarkers and echocardiography parameters was also evaluated in patients with heart failure with preserved ejection fraction. The cardiac biomarkers such as NT-proBNP, hsTnT, and high-sensitivity troponin I (hsTnI) were used [5]. The levels of hsTnT and NT-proBNP in RA patients were examined and correlated to healthy age-matched and sex-matched patients. It revealed hsTnT and NT-proBNP elevation in RA patients independent of cardiovascular (CV) risk factors [7]. In a study conducted, it was revealed that hsTnT and NT-proBNP are poorly related to cardiac anatomy and function in RA patients. However, the hsTnT and NT-proBNP levels found in the study population were moderately elevated compared to what was found in the common population. Hence, this supported the mechanisms of cardiac marker fluctuations in RA patients [6]. Anti-apo lipoprotein A-I (anti-Apo A-I) IgG antibodies have themselves also been shown to be a good predictor of subsequent major CV events (MACE) in patients with rheumatoid arthritis. Elevation of levels in anti-Apo A-I indicates the presence of atherosclerotic plaques in humans. NT-proBNP is a promising cardiac biomarker known to be linked to ventricular dysfunction and cardiac ischemia. In a cross-sectional study, it was revealed that NT-proBNP levels were increased in RA patients as compared to the control population, and this was also linked with many cardiac disease outcomes. NT-proBNP was predictive of subsequent MACE, but it did not enhance the traditional predictive CV risk factors. Oxidized low-density lipoprotein (ox-LDL) was proposed to be used as a promising biomarker for CVD in RA due to its role in atherogenesis and its release from the inflamed rheumatoid joint. Ox-LDL was also known to be related to CVD in the general population, and it could be used independently to assess the disease activity in RA and subclinical atherosclerosis patients. In a study, the added predictive ability of the biomarkers was examined for the enhancement of the global AUC by adding the biomarkers to the FRS. It was observed that the univariate prognosis ability of ox-LDL, NT-proBNP, or anti-Apo A-I was present, but when merged with the traditional risk factors (FRS), only anti-Apo A-I improved the discrimination of the FRS [9].

hsTnT showed a greater association with cardiac structure and function than NT-proBNP. This was explained in a previous study, which revealed that hsTnT revealed a relationship to an abnormal myocardium irrespective of the cause, while NT-proBNP was mainly associated with a compensatory cardiac ventricular response to pressure and volume overload. Another study previously revealed that the release of hsTnT in patients was associated with high LVMI and microvascular dysfunction, leading to an ischemic myocardium [6]. Among RA patients, it was observed that in patients who experienced an increase in inflammation, there was an increase in biomarkers associated with subclinical myocardial injury. This could further suggest the examination of the benefit of using hs-TnT for CVD risk stratification in RA [15]. High-sensitivity cardiac-specific troponin-I (cTn-I) was found to be higher in patients with RA as compared to controls. High-sensitivity cTn-I concentrations were found to be increased in patients with RA without heart failure, independent of the inflammatory markers or the cardiovascular risk profile. Such an increase in the concentration could indicate subclinical, indolent myocardial injury [17].

RA patients who have increased systolic blood pressure, used NSAIDs, or had positive APCA with increased hsTnT levels, indicating a greater risk of LVMI, will help physicians pay greater attention to clinical observation and cardiac biomarkers in RA patients before the clinically overt cardiac disease comes forward [6]. A longitudinal study and a cross-sectional study were done, which indicated an association between markers of inflammation, RA disease activities, and medication used in the treatment. It suggested that CRP levels are associated independently with NT-proBNP [18]. A study confirmed that BNP was raised in patients with RA and suggested that the significant rise was occurring despite identical LV mass and function to blood pressure and age-healthy volunteers. It suggested that a significant relationship is present between hs-CRP levels and LVMI in patients with RA. It indicated a significantly relevant relationship between inflammation evaluated by hs-CRP and BNP [19].

Some researchers have also suggested the use of advancements in medical imaging for early and accurate CV risk stratification compared to conventional CV risk calculators [14]. However, further studies are needed before its application.

However, there are some limitations to using cardiac biomarkers. hsTnT elevation in RA patients could be associated with stunned or hibernating myocardium and apoptosis or necrosis of myocytes secondary to subendothelial ischemia. The rise in circulating hsTnT was also linked to a multivariate analysis of obesity, according to previous reports that demonstrated an association between hsTnT, obesity, and metabolic syndrome. CRP levels were also associated with hsTnT concentrations [7]. There was a lack of association between the disease activity or rheumatic fever and the differential use of antirheumatic therapy in the patients. Further validation is needed before using these biomarkers in routine practice [9]. No information about the long-term outcomes was found in the cross-sectional study. We could not eliminate the possibility of inflammatory mediators affecting the hs-cTn-I concentrations [17]. There is a level of uncertainty and ambiguity when it comes to the cardiac troponin elevation, as encountered in some patients who do not present with ischaemic symptoms, and this could lead to misdiagnosis at times [20].

A randomized control trial was done to study the effect of using IL-6 receptor blockers like tocilizumab along with disease-modifying anti-rheumatic drugs (DMARDS) on decreasing the cardiovascular risk in patients with rheumatic arthritis. The cardiac biomarkers were assessed in order to evaluate the risk in rheumatic arthritis patients. It was observed that the cardiac biomarkers, namely NT-proBNP and hsTnT, were elevated before tocilizumab was administered, indicating a cardiovascular risk. Later, the study concluded that on the administration of tocilizumab, there was a decrease in the level of cardiac biomarkers, indicating a lowering in the CV risk [21].

However, it can be noted that the combined approach of using cardiac biomarkers along with the traditional CV risk factors (FRS) could impact improving medical decision-making and help in the better prognosis of the patient, and this could be an innovative mode for screening of cardiovascular events for patients with RA [9].

Limitations

Our literature review has some limitations. We have limited our analysis to English articles that were published within the last 11 years, precisely choosing those at least 13 years old. We have also used only free articles, and our review was limited to databases such as PubMed, Medline, and Google Scholar. More research would be needed for specific conclusions.

## Conclusions

Our research has highlighted the importance of evaluating rheumatoid arthritis based on cardiac biomarkers. It emphasized how it can, in the future, provide a better prognosis and effectively improve risk stratification. Although some challenges tend to exist, further research and evaluation could help in improving the possibility of using cardiac biomarkers for evaluating cardiovascular risk in rheumatoid arthritis patients. These could be added to the traditional risk factors (FRS) in the future, thereby making it easier for clinicians in the future. Nonetheless, large-scale RCTs are required in order to organize these methods for better diagnostic and prognostic advancements in the future medical field.
